# Recent development in the preservation effect of lactic acid bacteria and essential oils on chicken and seafood products

**DOI:** 10.3389/fmicb.2022.1092248

**Published:** 2022-12-23

**Authors:** Heena Sharma, Hafize Fidan, Fatih Özogul, João Miguel Rocha

**Affiliations:** ^1^Food Technology Lab, Dairy Technology Division, ICAR-National Dairy Research Institute, Karnal, India; ^2^Department of Tourism and Culinary Management, University of Food Technologies, Plovdiv, Bulgaria; ^3^Department of Seafood Processing Technology, Faculty of Fisheries, Çukurova University, Adana, Türkiye; ^4^LEPABE – Laboratory for Process Engineering, Environment, Biotechnology and Energy, Faculty of Engineering, University of Porto, Porto, Portugal; ^5^ALiCE – Associate Laboratory in Chemical Engineering, Faculty of Engineering, University of Porto, Porto, Portugal

**Keywords:** chicken products, seafood, bioactive compounds, essential oils, lactic acid bacteria, action mechanisms, preservation, biological activity

## Abstract

Chicken and seafood are highly perishable owing to the higher moisture and unsaturated fatty acids content which make them more prone to oxidation and microbial growth. In order to preserve the nutritional quality and extend the shelf-life of such products, consumers now prefer chemical-free alternatives, such as lactic acid bacteria (LAB) and essential oils (EOs), which exert a bio-preservative effect as antimicrobial and antioxidant compounds. This review will provide in-depth information about the properties and main mechanisms of oxidation and microbial spoilage in chicken and seafood. Furthermore, the basic chemistry and mode of action of LAB and EOs will be discussed to shed light on their successful application in chicken and seafood products. Metabolites of LAB and EOs, either alone or in combination, inhibit or retard lipid oxidation and microbial growth by virtue of their principal constituents and bioactive compounds including phenolic compounds and organic acids (lactic acid, propionic acid, and acetic acid) and others. Therefore, the application of LAB and EOs is widely recognized to extend the shelf-life of chicken and seafood products naturally without altering their functional and physicochemical properties. However, the incorporation of any of these agents requires the optimization steps necessary to avoid undesirable sensory changes. In addition, toxicity risks associated with EOs also demand the regularization of an optimum dose for their inclusion in the products.

## 1 Introduction

Chicken meat and seafood are recognized as nutritionally superior foods with lower fat content and more unsaturated fatty acid content than meat from other species (Kumar et al., 2015b). Apart from containing proteins and amino acids with high biological value, vitamins, and other essential nutrients, these are economically affordable and not associated with any cultural or religious taboos owing to which chicken meat and seafood are consumed at a leading rate across the globe. However, the quality and nutritional value of the chicken and fish meat and the resultant products are of paramount importance which can influence its acceptability significantly. Furthermore, chicken and seafood also act as major potential sources of foodborne illness as they harbor various foodborne pathogens including *Salmonella* spp., *Escherichia coli*, *Listeria monocytogenes*, and *Campylobacter* sp. *Salmonella* infection has reported almost 94 million illnesses and 1.5 lakhs deaths worldwide (Heredia and García, 2018). Therefore, all the factors beginning from farm management to the table of the consumers should be emphasized and given due consideration in order to ensure desirable and optimum meat quality. The importance of ensuring food safety is increasing day-by-day. In this direction, various measures have been taken, such as the widespread use of chemical preservatives in food, to prevent the spread of pathogenic microorganisms in the food industry, extend the shelf-life of food, and prevent economic losses (Françoise, 2010). The association of these chemical preservatives with cancer and other health-related risks has necessitated researchers to explore safer alternatives including bio-preservation and the use of protective cultures. In this regard, a group of lactic acid bacteria (LAB) and their antimicrobials and essential oils (EOs) have been successfully explored for their multipurpose use in chicken meat and seafood. Generally, homofermentative LAB is considered for its meat preservation action owing to the production of only lactic acid from various sources of carbohydrates. LAB antimicrobials, such as bacteriocins (bioactive cationic peptides or proteins) and other organic acids, help in inhibiting spoilage and maintaining the quality of chicken meat and seafood (Ibrahim et al., 2021), while EOs are the secondary metabolites of plant origin that not only inhibit the pathogenic microorganisms but also pose several health benefits including antimicrobial and antioxidant activity (Sharma et al., 2020). However, owing to the unique composition of meat and seafood and the different nature of EOs and LAB, interaction among these results in several complex mechanisms leading to either synergistic or antagonistic actions. Looking at the potential of the use of LAB and EOs in the meat industry especially, chicken meat and seafood, the present review summarizes the basic principles and mechanisms of action of different EOs and LAB metabolites in meat and seafood preservation. Furthermore, this review is aimed at updating the knowledge regarding various active components of EOs and different LAB strains used in the chicken meat and seafood industry. The detailed discussion has also been focused on the significance and health benefits of EOs.

## 2 The essential parameters of chicken and seafood products for human consumption

### 2.1 Nutritive value of chicken meat and seafood

Chicken meat and meat products have an edge over other types of meat in terms of nutritional value offered to consumers. Scientific studies validate the presence of higher protein and lower fat content in chicken meat than in red meat, thereby giving a feeling of satiety to consumers. Noteworthy, it is recommended to consume it without skin as the latter contains almost 3 times more fat than chicken meat without skin. Furthermore, the lower content of saturated fatty acids in chicken meat results in lower calorific value, thus making it suitable for people suffering from cardiovascular diseases. The risk of such diseases could be reduced by 19% upon replacing red meat with chicken meat in diets (Bernstein et al., 2010). Reports also suggest a positive correlation between dietary saturated fatty acid content and insulin resistance, thereby the occurrence of diabetes (Pan et al., 2011). Therefore, chicken meat can be considered a boon for people afflicted with diabetes as compared with red meat. However, cholesterol content does not contribute to any striking feature of the nutritional value of chicken meat as it is present in almost the same content as in other types of meat. The availability of easily degradable and high-quality proteins in chicken meat makes it a valuable and healthy source of animal protein not only for people with no specific requirement (average daily requirement is 0.66 g kg^–1^ bodyweight) but also for pregnant women (23 g day^–1^ more protein required during late gestation period), athletes, and young children (1.2–1.3 g kg^–1^ body weight) as their requirement is at par with other people. Moreover, the lower level of collagen further adds to the easier digestible characteristic of chicken meat. Among vitamins and minerals, chicken meat contains higher amounts of calcium, sodium, phosphorous, and vitamins B_3_ (niacin), A, and B_6_ than other meats (Talukder et al., 2013). These minerals and vitamins play vital roles in various physiological activities such as protein synthesis, energy metabolism, maintaining lower lipids, cholesterol levels, and normal gut functions. Though the mechanism is still unclear, the production of aromatic amines, N-nitrous compounds, and others are directly involved in inducing cancers in humans with the consumption of red meat while, white meat is often associated with a lower incidence of cancer (Zhu et al., 2014). Thus, it can be inferred that the availability of all the essential nutrients confers nutritional benefits to chicken meat; however, research based on the impact of chicken consumption on health effects should be interpreted cautiously as human physiology is a complex process involving various significant factors influencing the occurrence of certain diseases.

As compared to red and white meat, meat obtained from fish and seafood is considered to be more nutritious and healthier for malnutrition and obese people (Cena and Calder, 2020; Maulu et al., 2021). Globally, fish contributes to the third most important source of animal proteins after cereals and milk and contains higher protein content and lesser energy as compared to red meat. The health benefits associated with the consumption of fish and seafood also provide them an edge because they are nutritious and healthy foods for human consumption (Tacon et al., 2020). Omega-3 polyunsaturated fatty acids present in seafood make them suitable for persons more prone to cardiovascular diseases. Furthermore, the trace minerals, such as iodine, chromium, and selenium, required for maintenance and daily activities are also present in abundance in seafood.

### 2.2 Quality parameters to assess chicken meat quality and seafood

Though it is widely accepted that chicken meat and meat products are among the top-most commodities consumed throughout the world, there are various technological and quality parameters that influence their acceptability and purchasing (Kumar et al., 2015a). The pH, water holding capacity, drip loss, and color of the meat significantly impact the meat quality and its subsequent shelf-life owing to various factors including stress before slaughter, the genotype of the animal, and meat handling and processing conditions. Animals subjected to chronic stress before slaughter often produce meat that has higher ultimate pH (6.5–6.8). Though higher pH inhibits drip loss and presents a dry appearance on the meat surface, such type of meat, also known as dark, firm, and dry, is more prone to microbial spoilage and thus, ultimately has a shorter shelf-life (Gonzalez-Rivas et al., 2020). On the other hand, higher temperatures and lower ultimate pH of meat cause protein denaturation, thereby decreasing the water-holding capacity. Exposure to heat stress brought a significant increase in creatine kinase and glutamic-pyruvic transaminase of chicken plasma associated with higher cooking loss and lower water holding capacity. Furthermore, lower pH is also associated with the oxidation of myoglobin (meat pigment; purple color) to met-myoglobin (brown meat color) which is aesthetically unappealing (Pardo et al., 2021).

Due to their highly perishable nature, the majority of the seafood is transported in ice across various parts of the country. Higher moisture content and neutral pH of seafood also make them more prone to microbial spoilage (Yavuzer and Köse, 2022). Microbial growth generally leads to the production of alkaline compounds in fish, including ammonia, which results in a higher pH value (9.2) of seafood, thereby indicating incipient spoilage conditions. Furthermore, the degradation of proteins also results in the development of amino acids and other amines, as a result of which the total volatile base nitrogen of seafood increases. It has been considered that a level of 30–35 mg TVBN per 100 g is acceptable as the average quality of fish. Furthermore, the production of histamine, cadaverine, and putrescine, as detected by analytical techniques, suggests possible signs of spoilage in fish (Yavuzer, 2021). Therefore, careful handling and storage of seafood is a must for its improved shelf-life and better sensory acceptability.

## 3 The significance of lactic acid bacteria and their preservative effects on chicken and seafood products

### 3.1 Bio-preservative properties

Foodborne infections and intoxications are significant problems due to their harmful effects on human health and the economy. Despite advances in food technology, the number of illnesses caused by unsafe food is increasing rapidly. The past several decades have witnessed the wide-scale utilization of chemical preservatives in the food industries. However, several studies have shown that these chemical food preservatives are associated with toxicological problems and diseases (allergic reactions, heart disease, neurological problems, and cancer). Therefore, interest in replacing chemical preservatives with natural alternatives that are safer for consumers and the environment has increased over the past decade. Additionally, today’s consumers worldwide are more health conscious and consume foods that do not contain artificial preservatives and use bio-preservatives (Alipin and Safitri, 2016).

Biopreservation is defined as the preservation of foods using biological agents or extending the shelf-life of foods and improving food safety by using microorganisms and their metabolites. It refers to the inhibition of unwanted or pathogenic microorganisms due to antagonistic microbial interference for nutrient competition and the production of antimicrobial metabolites, such as organic acids, hydrogen peroxide, diacetyl, reuterin, bacteriocins, and other low-molecular-weight metabolites (Sakaridis et al., 2014). Over the past few decades, LAB has been widely used for the preservation of fermented and cooked meat products, and a variety of strains have been found to be effective against pathogens and spoilage microorganisms (Bartkiene et al., 2020, 2022; Küley et al., 2020; Zokaityte et al., 2020; Ağagündüz et al., 2021, 2022; Sharma et al., 2021; Petkova et al., 2022; Rathod et al., 2022; Trakselyte-Rupsiene et al., 2022; Yilmaz et al., 2022a,b). However, research on the biopreservation of fresh poultry meat is quite limited and is even scarcer for fresh fish products. In food production, applying a minimum level of processing and preferring natural additives to ensure food safety is essential. For this purpose, antagonistic microorganisms and their antimicrobial metabolites and the use of biological control systems that induce the inhibition of spoilage bacteria are recommended. Antimicrobial compounds are used in food because of their ability to slow down the development of undesirable microorganisms in food products (Kupryś-Caruk et al., 2019).

Nevertheless, bioprotective culture is defined as microorganisms that prevent the proliferation of pathogens that cause food spoilage and reduce the shelf-life of foods, and this process is carried out without changing the organoleptic properties of foods. The best-known of these microorganisms are LAB and Gram-positive bacteria (Djenane et al., 2006; Guluwa et al., 2021). Several researchers investigated the lactic acid bacteria and their preservation properties on poultry and fish products (Erol et al., 2021; Dissasa et al., 2022). For example, a potential natural food additive using LAB from fermented *Tilapia niloticus* combined with different spices (9% turmeric, 6% chili, and 9% black pepper) on foodborne pathogens was studied. The greatest antimicrobial activity by LAB on *Bacillus cereus* was observed in fermented tilapia combined with black pepper. In contrast, fermented fish combined with chili demonstrated the greatest antimicrobial activity on *Staphylococcus aureus, Escherichia coli*, and *Salmonella enterica* Serovar Typhimurium (Erol et al., 2021). In another study, Dissasa et al. (2022) reported the antimicrobial activity of the antimicrobial peptides from LAB on the growth of pathogenic bacteria from fish in Ethiopia. With the well diffusion assay, *Lactiplantibacillus plantarum*, *Lacticaseibacillus casei* LC2W, and *Lacticaseibacillus paracasei* sub *paracasei* demonstrated strong antibacterial activity on *Edwardsiella tarda* (19 mm), *Aeromonas hydrophila* (18 mm), and *Pseudomonas fluorescens* (18 mm), respectively. The research demonstrates the antimicrobial activity of four strains of LABs on bacterial fish pathogens and states the application opportunities as a biopreservative of fish products.

Poultry meat is thought of as one of the most common foods that result in foodborne infection and intoxication. Alipin and Safitri (2016) reported the potential of LAB from a healthy broiler intestine to prevent the growth of *Salmonella* spp. (*Lactobacillus* spp., *Streptococcus* spp., and *Bifidobacterium bifidum*). Sakaridis et al. (2014) studied the possibility of *Ligilactobacillus salivarius* being used as a protective culture to enhance the safety and prolong the shelf-life of chicken products. They found the antagonistic activity of LAB from poultry carcasses against the pathogenic bacteria (*Salmonella* spp. and *L. monocytogenes*). They reported a decrease in the *Salmonella* population on the fifth day. Wyszyńska and Godlewska (2021) studied strategies to prevent the development of *Campylobacter* in the microbiome of chickens using LAB, which appears to represent a major barrier against pathogen invasion in the gastrointestinal tract.

Lactic acid bacteria have many benefits for the food industry and find application in the preservation of food raw materials by participating in the optimization of the metabolic activity of the microbial environment and reducing the viability of the unwanted microbial population (Abiola et al., 2022). Masoumi et al. (2022) studied the microbial and physicochemical properties of raw chicken filets immersed in yogurt containing *L. casei* stored at 4°C for 9 days. They revealed that the amount of *S. aureus*, fecal coliforms, yeast, and mold (filamentous fungi) counts decreased in chicken filets preserved with the probiotic yogurt. Petkova et al. (2022) reported that LAB, a major component of fermented foods, is a major biological tool for a wide variety of food toxins, including bacterial toxins (Shiga toxin, listeriolysin, and botulinum toxin), mycotoxins (aflatoxin, ochratoxin, zearalenone, and fumonisin), pesticides (organochlorines, organophosphates, and synthetic pyrethroids), and heavy metals and natural anti-nutrients including phytates, oxalates, and cyanide-generating glycosides.

### 3.2 Preservation mechanism by using LAB antimicrobials

Although many microorganisms have a bioprotective effect, almost all are LAB. The most commonly used lactic acid bacteria are the bioprotective cultures *Aerococcus, Carnobacterium, Lactobacillus, Bifidobacterium, Lactococcus, Enterococcus, Leuconostoc, Oenococcus, Pediococcus, Melissococcus, Tetragenococcus, Vagococcus, Streptococcus*, and *Weissella*. LAB has effective bioprotective potential and inhibitory effects, and this is because it naturally dominates the microbiota of many foods during storage. In addition, it has competitive properties against pathogenic microorganisms when consuming food and can produce compounds, such as bacteriocin, organic acid, hydrogen peroxide, and enzymes. In addition, antimicrobial peptides produced by LAB are easily degraded by digestive proteases and therefore do not cause disturbances in the gut microbiota (Khorshidian et al., 2016).

**FIGURE 1:**
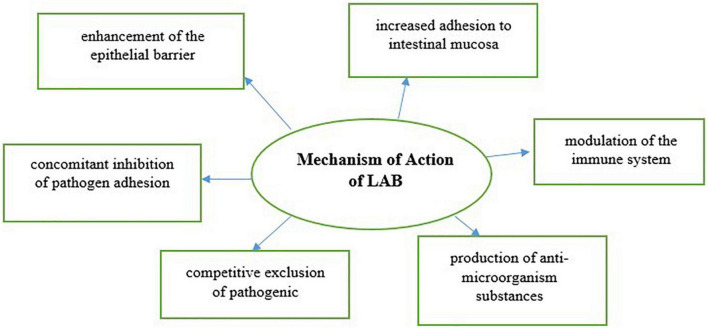
Mechanism of action of lactic acid bacteria.

The use of antimicrobial substances produced by LAB leads to a modification of the environment to win the competition against microorganisms. The antimicrobial activity of LAB is performed by the compounds produced by them, which could be grouped as organic acids (mainly, lactic acid, and acetic acid), diacetyl, hydrogen peroxide, reuterin, and bacteriocins, which also show antimicrobial activity in microorganisms that affect food safety and shorten their shelf-life. The mechanism of the action of lactic acid bacteria is presented in Figure 1. For example, lactic acid lowers the medium’s pH and increases the cell membrane’s permeability. In this way, it enhances the action of other antimicrobial substances. In addition, due to its hydrophobic characteristic, the undissolved form of lactic and acetic acid enters the cell by passing through the cell membrane. Cell death occurs due to its solubilization in the cell and a decrease in cytoplasmic pH. LAB forms hydrogen peroxide (H_2_O_2_) with the enzyme flavoprotein oxidase in the presence of oxygen. Since there is no catalase enzyme in LAB, the H_2_O_2_ molecule gets accumulated in the environment and oxidizes the lipid membrane and cellular proteins of the target cell. Thus, it produces an antagonistic effect against bacteria, yeasts, molds, and viruses. The antimicrobial effect of the H_2_O_2_ molecule in non-lethal doses occurs as a result of its reaction with the thiocyanate compound in the presence of the lactoperoxidase enzyme found in milk.

Lactic acid bacteria produce antibacterial peptides or proteins, known as bacteriocins, composed of amino acids synthesized in the ribosomes of some LAB, that are released extracellularly to inhibit the growth of pathogenic microorganisms resistant to conventional antimicrobials. In addition, bacteriocins are versatile antimicrobial agents that can be used as biopreservative and can benefit the digestive system and health. Nisin and natamycin (pimaricin) are generally recognized as safe (GRAS) bacteriocins. Nisin is the most studied of the bacteriocins used in the food industry in terms of biochemistry and genetics. Nisin, an antimicrobial substance, is a polypeptide bacteriocin formed during the fermentation of modified milk that shows acidic properties. It shows greater temperature resistance between pH 3 and 7. Although it is effective against some Gram-positive bacteria and some spore-producing bacteria, it is not effective against molds, yeasts, and Gram-negative bacteria (Raman et al., 2022). Bacteriocins have different mechanisms of action. Some of them have the ability to increase the permeability of the cell membrane of the target microorganism by forming pores and can also inhibit the synthesis of the cell wall. Some manage to penetrate the cytoplasm of the bacterium and secrete DNA or RNA. Bacteriocins have a narrow range of inhibitory activity and can inhibit only strains closely related to the producing organism or inhibit a wide range of Gram-positive microorganisms. Bacteriocins used for biodefense can be incorporated into food in three ways: (i) inoculation of bacteria into the food, (ii) addition of purified or partially purified bacteriocins to the food, and (iii) the addition of a component fermented by bacteriocin-producing strains (Betancur-Hurtado et al., 2022).

### 3.3 Applications of LAB antimicrobials on chicken products

Lactic acid bacteria are considered natural microbiota of fermented meat products and exert their beneficial and preservative effect on meat and meat products owing to the production of bacteriocins. Bacteriocins are biologically active substances that are synthesized by ribosomal proteins and possess similar peptide structures (Silva et al., 2018). Among different classes of bacteriocins, class I belongs to the group involved in food preservation and is considered to tolerate high temperatures (Bolívar-Monsalve et al., 2019), while a purified class II bacteriocins, leucocin A, is recognized as a promising candidate with the potential to inhibit listerial activity and tolerate extreme conditions during the fermentation process in meat products (Barcenilla et al., 2022). For example, nisin produced from *Lactococcus lactis* is a class I bacteriocin that is used commercially by the food industry. On the other hand, lantibiotics (consisting of B α-peptide and type A1 β-peptide) exhibit biopreservation activity as they form pores in the cell membrane of the bacteria. Though a few of the bacteriocins are recognized as inhibitors of both spoilage and pathogenic organisms, the majority of these exert an inhibitory effect on Gram-positive bacteria (Yildirim et al., 2016; Caulier et al., 2019). Since bacteriocins need to cross the cell wall for exerting their effect on Gram-negative bacteria, certain physical and chemical treatments are required for the synergistic approach. However, the presence of bacteriocin-specific receptors on cell membrane protein might make the bacteriocins execute their mode of action (Acuña et al., 2012). Other compounds that are generated during the fermentation process of meat and seafood products by LAB comprise aldehydes, ketones, organic acids, acetoin, and hydrogen peroxide, which possess immense antimicrobial and antifungal properties (Egan et al., 2016). Various pathogenic organisms, such as *L. monocytogenes*, *Salmonella* spp., and *E. coli*, in chicken and seafood, have been targeted by the application of LAB. The bio-preservative effect of LAB bacteria has been elucidated in chicken products by decreasing the growth of *L. monocytogenes* and *Salmonella* by 85 and 92%, respectively, during the refrigerated storage of 6 days (Sakaridis et al., 2012). Since the incorporation of LAB might alter the sensory properties of chicken products owing to the reduction in pH, studies demonstrate no decrease in the overall acceptability of chicken and seafood (Balay et al., 2017). In another study, *Leuconostoc pseudomesenteroides* decreased pathogen counts to 0.9 log CFU/g under MAP-packaged fresh chicken meat burger (Melero et al., 2012). Though research on the bio-preservative effect of LAB suggests that metabolites produced by LAB certainly possess antimicrobial characteristics and have the potential to improve the shelf-life of chicken products without alteration of sensory properties, considering the fact that meat matrix is a complex food system as a result of which reproducible results cannot be expected.

### 3.4 Synergistic effect of LAB antimicrobials with other compounds

Due to synergistic effects, some authors demonstrated that combined treatments of lactic acid bacteria and other compounds could have better antimicrobial activity than either treatment (Ruíz et al., 2012). According to the literature, the lactic acid bacteria could be combined and can demonstrate their synergistic potential with various compounds (Zhou et al., 2016). For example, Ruíz et al. (2012) reported the extensive inhibitory potential of a bacteriocin-like substance from *Lacticaseibacillus rhamnosus* L60 and *Limosilactobacillus fermentum* L23 on pathogenic bacteria. In contrast, Aminnezhad et al. (2015) revealed the synergistic ability of the cell-free substances of *L. rhamnosus* and *L. casei* against the growth of *Pseudomonas aeruginosa* PTCC 1430.

The antimicrobial activity of EOs is largely examined. Hashem et al. (2019) showed that the combination of lactic acid bacteria with EOs led to a synergistic activity against *S. aureus*. Almost all studied cell-free supernatant–medicinal oil combinations showed marked synergistic activity against *S. aureus, E. coli*, and *Klebsiella pneumoniae*. In another study, Kim et al. (2022) reported the synergistic antibacterial potential of lactic acid bacteria combined with *Curcuma longa* rhizome extract against *Cutibacterium acnes*.

Lactic acid bacteria could be used in synergism with other compounds in order to reduce pathogenic bacteria in plants, too. In the study of Lin et al. (2020), it was reported that the ability of *Lactiplantibacillus pentosus* and *Leuconostoc fallax* to suppress plant diseases is highly dependent on chitosan. *L. pentosus* and *L. fallax* together with chitosan expressed strong inhibitory effects against plant pathogens. LAB strains are in a desirable synergism with the mixture of glycerol, sucrose, and chitosan after being cultured in fish surimi due to their ability to inhibit the growth of *Alternaria brassicicola* and black rot caused by *Xanthomonas campestris* pv. *campestris*, known to cause vegetable diseases including cabbage black spot and black rot.

However, antibiotic-resistant microorganisms are a common health concern that reported the positive synergistic potential of bacteriocins from lactic acid bacteria isolated from traditionally fermented products of India with antibiotics, including cefotaxime, polymyxin B, imipenem, and tigecycline, against pathogens, such as *Streptococcus pyogenes, Enterococcus faecalis, Escherichia coli, Klebsiella pneumoniae*, and *Bacillus cereus*.

Lactic acid bacteria find applications in a combination with organic salts. According to Bolivar et al. (2018), the organic salts namely butyrate, propionate, succinate, citrate, formate, fumarate, glutamate, and acetate could be used in combination with probiotics in order to inhibit the growth of *Vibrio alginolyticus, A. hydrophila, E. coli, P. aeruginosa*, and *Streptococcus agalactiae*. The lactic acid strains show their antibacterial capacity combined with mineral elements. Gyawali and Ibrahim (2012) reported that copper in combination with lactic acid has been shown to eliminate the presence of foodborne pathogens, such as *Salmonella* and *E. coli* O157:H7.

## 4 The preservative effects of EOs on chicken and seafood product

Interest in spices and aromatic and other plants, which are natural sources of antioxidants, has increased in recent years (Aboukhalaf et al., 2020, 2022; Neves et al., 2021; Mechqoq et al., 2022; Melo et al., 2022; Munekata et al., 2022; Özogul et al., 2022). Natural plant extracts are used in the food industry as alternative preservatives. It is possible to prevent or delay some chemical spoilage that occurs during food storage with the use of small amounts of EOs that are potential antioxidants due to their radical scavenging activities (Dupuis et al., 2022). Mariotti et al. (2022) confirmed the importance of evaluating natural products to consolidate the idea of safe EO applications in reducing and preventing intensive livestock infections.

Essential oils could be produced by different parts of plants. They consist of naturally occurring antimicrobial and antioxidant agents, which are extracted from different plant materials, such as leaves, bark, stems, roots, flowers, and fruits. Although more than 3,000 types of EOs are known, only some of them are of commercial interest for applications in the food industry.

### 4.1 Antimicrobial activity

Essential oils are important agents with anti-inflammatory, antibacterial, and antioxidant properties (Nehme et al., 2021) due to their potential to be an alternative to chemical preservatives and antibiotics, especially as a preservative against foodborne pathogens (Evren and Tekgüler, 2011). Bacteria, such as *L. monocytogenes, E. faecalis, Staphylococcus* spp., *Micrococcus* spp., *Bacillus* spp., *Campylobacter jejuni, Vibrio parahaemolyticus, Pseudomonas fluorescens, Shigella coli*, and *Escherichia* spp., and yeasts that cause food spoilage and poisoning can be destroyed with EOs. Gram-negative bacteria are said to be more resistant to the influence of EOs than Gram-positive bacteria, and this resistance of Gram-negative bacteria may arise from the cell wall. The antimicrobial properties of plant EOs are related to the main bioactive components in the composition of the plant sample, such as phenolic acids, terpenes, aldehydes, and flavonoids. The cell permeability and sensitivity could be affected by several mechanisms, such as changing the fatty acid profile, influencing the structure of cell membranes, and destroying membrane proteins. The aromatic and phenolic compounds manifest their antimicrobial ability due to the structural and functional transformation that are provoked in the bacterial cytoplasmic membrane (Yousefi et al., 2020).

Several studies have proved the influence of EOs on the quality and shelf-life of chicken and fish products. For example, Sharma et al. (2017) revealed that the fresh chicken sausages vacuumed and stored for 45 days in combination with clove, basil, cassia, and thyme oils showed a slower rate of increase in microbial count than the control sample. In another study, Sharma et al. (2020) revealed that chicken samples treated with EOs were characterized by a lower microbial count and, therefore, enhanced the shelf-life of chicken sausages. Alhijazeen (2021) reported the positive antimicrobial effect of the oregano essential oil and rosemary extract on the survival and growth of *S. aureus* and the total aerobic bacteria in cooked ground chicken meat stored at different temperatures. Wei et al. (2022) stated that cassia bark EO, cinnamon EO, tea tree EO, and angelica EO had the best antibacterial effect treated on marinated chicken. According to the results of Zubair et al. (2022), clove and cinnamon EOs extended the shelf-life of the studied products as they showed significant antimicrobial properties for total plate count and *Salmonella* during storage for 45 days of ready-to-eat chicken sausages.

The effect of EOs is investigated also in a combination with other current culinary treatments. For example, “sous vide” is considered a cooking method for vacuumed products placed in a water bath or steam oven at controllable low temperatures and is used for the treatment of various types of meat and vegetables. In ordinary boiling water, nutrient-rich food loses a great deal of them as they are passed into the fluid. Unlike the traditional cooking methods, foods could be prepared through the method of “sous vide,” where they retain almost all their nutritive value. Kacaniova et al. (2021) studied the sous vide thermal treatment on the microbiological quality of fresh turkey breast meat after the products were processed with thyme and rosemary Eos. The study showed that the sous vide method with the combination of EOs is an effective method and it can be used to protect the microbiota of turkey meat and *L. monocytogenes* survival. According to Hien and Dao (2021), while EOs are used and added to food products, they are not very effective in their pure form. They attributed the reason for the lower effectiveness of the pure oils to the lipophilic nature of the oils and the difficulties caused by this when used in food products. Therefore, they utilized nanoemulsions obtained from black pepper essential oil characterized by good dispersion, long-term stability, and transparency. Their study showed that *E. coli* and *S. enterica* were more sensitive to black pepper essential oil nanoemulsion than free essential oil.

Essential oils find application in different forms of edible coatings used widely in the food industry in order to extend the shelf-life of food products. In a study, it was shown the antimicrobial ability of the milk-protein-based edible films containing oregano, pimento, or a mix of oregano–pimento EOs on muscle slices against *E. coli* O157:H7 or *Pseudomonas* spp. (Oussalah et al., 2007).

Essential oils are also used as growth promoters of livestock, due to the fact that they find application as feed additives to improve feed intake, and to improve animal health status (Mucha and Witkowska, 2021). Oblakova et al. (2021) observed that the addition of *Matricaria chamomilla, Rosmarinus officinalis, Lavandula angustifolia, Origanum vulgare, Thymus vulgaris*, and *Hypericum perforatum* EOs to the feed of turkey broilers had a positive influence on the microbial quality of breast and thigh meat.

However, fish meat is one of the food products that is highly perishable due to its composition (Hassoun and Çoban, 2017). In the study of Kunova et al. (2021), the EOs from *Citrus lemon* and *Cinnamomum camphora* were used to treat the rainbow trout meat for evaluating the microbiological quality (viable counts of bacteria and identification of present microbiota). Their results showed that lemon and *C. camphora* EOs had a definite effect on the microbiological quality of fish meat, maintaining a high microbial quality of the fish filets during 7 days of cold storage. Al-Hajj et al. (2017) revealed that the *Pulicaria inuloides* EO inhibited all tested microorganisms, *viz*. *L. monocytogenes, E. coli*, and *S. aureus*. They also reported that *Pulicaria inuloides* and *Pulicaria crispa* EOs are able to eliminate *L. monocytogenes, E. coli*, and *S. aureus* inoculated in fish filets. According to Austin and Mcintosh (2006), the natural oregano essential oil had the ability to extend the shelf-life of rainbow trout containment showing good microbial load results. The results reported by Maghami et al. (2019) showed that coating fish filets with fennel EO significantly increased the microbiological safety of the product as they showed a lower number of mesophilic, psychotropic, pseudomonas, and lactic acid bacteria in coated fillets compared with control samples.

### 4.2 Antioxidant activity

A large proportion of plant EOs is classified by the US Food and Drug Administration (FDA) as GRAS (generally safe and harmless) additives that are applied to improve the organoleptic properties of foods or are used as food additives. Studies have been done that define their antibacterial, antiviral, antifungal, anti-inflammatory, antiseptic, antioxidant, anti-toxinogenic, and insecticidal properties. Studies have focused on the utility of these compounds in eliminating microorganisms that have become resistant to antibiotics. EOs are also potentially effective in food preservation.

Antioxidants are important substances in terms of nutrition as they reduce physiological stress in organs and cells. Disease resistance and immune competence in animals and humans are linked to the antioxidant mechanism. The compounds with the highest risk of oxidation are lipids. Oxidation of lipids occurs during the storage of raw materials, processing, heat treatment, and storage of finished products. Adding antioxidants, especially to oils and foods containing fat, is a method of extending shelf-life. Due to the suspicion that synthetic antioxidants may be carcinogenic, the use of antioxidants such as BHA (Beta Hydroxy acids) and BHT (Butylated hydroxytoluene) in foods has been restricted. In addition, due to the undesirable effects of oxidized lipids on the human body, the formation of these products in foods should be prevented as much as possible. Synthetic antioxidants are often used in food processing to extend the shelf-life of foods.

For this reason, research into using EOs as alternative antioxidants has accelerated in recent years. The antioxidant properties of EOs are due to the phenolic hydroxyl groups in the components’ structure. The antioxidant effect of these oils varies depending on the number of active ingredients, the type of solvent used in the extraction, and the extraction method.

In their study, Sharma et al. (2020) observed significantly better oxidative stability due to the higher values for DPPH (2,2-diphenyl-1-picrylhydrazyl) activity and total phenolic content for chicken products treated with four different blends of EOs. In their study, Albarracín et al. (2012) showed that the use of the thyme and rosemary EOs reduced the oxidative processes of tilapia filets from the ninth day of storage. According to Sharma et al. (2017), the treatment of chicken sausages with plat EOs showed the least rate of increase in oxidation.

The availability of phenolic compounds from EOs was evaluated by the determination of total phenolic compounds present in the films during storage (Oussalah et al., 2007). They evaluated the antioxidant properties of edible films treated with EOs during storage and demonstrated that oregano-based films had the ability to stabilize lipid oxidation in beef muscle samples, whereas pimento-based films presented the highest antioxidant activity. The results reported by Zubair et al. (2022) also showed the positive effect of essential oil, clove oil, on the polyphenol content and DPPH at a storage period of 45 days.

## 5 Combined impact of LAB and EOs on chicken and seafood

The group of lactic acid bacteria has been used widely in the meat industry to achieve the desired techno-functional properties and storage stability of chicken and seafood products. In addition to technological properties, these also impart characteristic sensory attributes to the meat products, as a result of which the consumer acceptability of the meat products is enhanced. The main function of LAB is to lower the pH of the meat product; however, various factors influence the final pH of the product, thus determining the safety and storage stability of the finished product. Being probiotic in nature, LAB also confers potential health virtues to meat products in addition to improving the techno-functional properties of meat products (Table 1). However, along with LAB, other microorganisms may also survive and grow under a conducible environment produced during the fermentation of meat products, which in turn, may pose a risk to human health. Therefore, a complete, safe, and wholesome meat for humans may include antimicrobials, which could selectively inhibit the growth of pathogenic or undesirable microorganisms.

**TABLE 1:** Application of LAB-produced bacteriocins and organic acids in chicken and seafood.

S. no.	Lab species	Metabolite	Preservative action	Food	References
1	*Lactococcus lactis*	Nisin	Bacteriocin	Trout	Wang et al., 2020
2	*Lactobacillus delbrueckii* subsp. *bulgaricus*	Lactic acid	Organic acid	Salmon	Ayivi et al., 2020; Jadhav and Annapure, 2021
3	*Lactococcus lactis* subsp. *cremoris*	Formic acid	Organic acid	Poultry	Gómez-García et al., 2019; Ricke et al., 2020
4	*Lactococcus lactis* subsp. *lactis*	Succinic acid	Organic acid	Chicken meat	Radkowski et al., 2018
5	*Limosilactobacillus reuteri*	Malic acid	Organic acid	Meat products	Ghoul and Mitri, 2016
6	*Lactococcus lactis* subsp. *lactis*	Propionic acid	Organic acid	Poultry products	Gonzalez-Fandos et al., 2020
7	*Lactobacillus acidophilus*	Acetic acid	Organic acid	Salmon	Gómez-García et al., 2019
8	*Lactobacillus acidophilus*	Butyric acid	Organic acid	Poultry	Reuben et al., 2021
9	*Limosilactobacillus reuteri*	Hydrogen peroxide	Antimicrobial	Chicken meat	Asare et al., 2020
10	*Streptococcus diacetyl lactis*	Diacetyl	Antimicrobial	Chicken meat	Yu et al., 2021

### 5.1 The preservative effect of LAB and EOs on chicken products

Chicken products have a shorter shelf-life as compared to red meat owing to their high polyunsaturated fatty acid content (Sharma et al., 2018). Therefore, it is suggested that the fermentation process can be used to enhance the shelf-life of chicken meat products with desired techno-functional and sensory properties. The majority of the fermentation processes are caused by the group of LAB, and it is considered that these have a higher chance of bacterial survival in the meat matrix than all other microorganisms. In this regard, the beneficial effect of EOs has been observed along with LAB in chicken meat products, wherein no inhibition effect was observed in the growth of LAB (Leite de Souza, 2021). The Italian-type sausage was incorporated with basil essential oil (0.75 mg g^–1^), and no change in the growth and survival of LAB was observed during 60 days of cold storage (Gaio et al., 2015). Similarly, no changes in viable counts were recorded in Tunisian dry fermented chicken sausage incorporated with a blend of oregano and thyme essential oil (0.25%) (El Adab and Hassouna, 2016), indicating that the combination of EOs and LAB has proven to be synergistic for extending the shelf-life of chicken products and inhibiting the growth of undesirable and spoilage bacteria. In another study conducted by Chaves-López et al. (2012), it was interesting to note that the incorporation of oregano essential oil (0.01%) in Spanish-fermented sausage enhanced the viable count of LAB over time. The increase in LAB count was attributed mainly to the diffusion of essential oil across the casing into the sausage matrix which could additionally exert stimulatory growth on LAB. EOs are known to exert a stimulatory effect due to the utilization of carbon and other energy sources (Badia et al., 2020). Furthermore, osmotic stressed conditions and potassium efflux contributed by EOs are well tolerated by LAB along with the generation of adenosine triphosphate, thus ensuring their survival even in the presence of EOs (Holley and Patel, 2005; El Adab and Hassouna, 2016). Therefore, these studies establish that LAB adapt well to the environment brought by the incorporation of EOs in the meat matrix, thus enabling this group of bacteria to outgrow other microorganisms.

Though several studies suggest that EOs have a stimulatory effect on the growth of LAB which, in turn, results in higher storage stability and shelf-life of chicken meat products, there are a few studies reporting that EOs alone could also improve and maintain the quality of chicken meat for a longer period of time. These findings have been observed in fermented chicken products, wherein no effect was observed on LAB viable count, while other pathogenic microorganisms including *S. aureus* and *L. monocytogenes* have been inhibited (Lu et al., 2015). In fact, fewer studies have reported the inhibitory effect of EOs on the growth of LAB bacteria in bologna sausage, dry sausage, and some ready-to-cook chicken products (Badia et al., 2020; Tomović et al., 2020).

### 5.2 The preservative effect of LAB and EOs on seafood products

Similar to chicken meat and meat products, seafood is also prone to microbial spoilage owing to its perishable nature due to higher moisture content. Generally, end products of enzymatic and microbial reactions in seafood often lead to the development of potential health-hazardous metabolic products which, in turn, deteriorate the physicochemical, sensory, and nutritional properties (Olatunde and Benjakul, 2018; Hussain et al., 2021). Therefore, suitable preservation techniques in terms of natural preservatives are required for keeping up the quality and storage stability, subsequently improving the shelf-life of seafood. In this regard, microorganisms, mainly LAB, and EOs have been used widely to preserve the natural aroma and quality of seafood such as bream filets *Megalobrama amblycephala* (Nisar et al., 2019), cobia steak *Rachycentron canadum* (Remya et al., 2017), shrimp *Parapenaeus longirostris* (Alparslan et al., 2016), and fish carp burger *Cyprinus carpio* (Ehsani et al., 2020). Though extensive literature is available regarding the beneficial effect of EOs on the microbial safety of seafood, the majority of the studies reported that the incorporation of EOs in fresh seafood including common carp filet (Zhang et al., 2017), red drum filet (Cai et al., 2015), and crayfish (Duman et al., 2015) inhibited the growth and decreased the viable count of LAB along with other microorganisms. However, sensory scores and shelf-life were enhanced by an average of 5–7 days for seafood. There are very few studies available on the effect of EOs on fermented seafood. Improvement in the production of fish-fermented flour was observed by the addition of *Pimenta racemosa* essential oil (0.5–2 μL g^–1^) in which the inhibitory effect was observed on the growth of pathogenic microorganisms; however, this study lacks the effect on the viable count of LAB bacteria (Adjou et al., 2021). In another study conducted by Boulares et al. (2018), sea bass filets were observed with lower total volatile base nitrogen content (1.12 vs. 30.47 mg 100 g^–1^) in the sample treated with lactic acid bacteria and citrus essential oil as compared with the control. In addition, biogenic amines including agmatine, putrescine, and cadaverine, were also decreased upon the combined application of LAB and essential oil, indicating the synergistic effect of these on the preservation of seafood.

## 6 Therapeutic benefits of LAB and EOs on chicken and seafood products

High moisture content, nutritive value, and neutral pH are considered to be significant factors playing a role in the quality and shelf-life of chicken meat and seafood products. Therefore, several preservative methods and techniques have been developed to maintain and improve their storage stability. However, owing to the carcinogenic and other harmful health effects associated with chemicals, suitable bio-preservative technologies have been investigated. Among these, the use of LAB and EOs have been proven successful owing to their contributions to the enhancement of the shelf-life and nutritive value of meat products without altering the sensory properties of the food (Kumar et al., 2018).

Lactic acid bacteria are the most important microbiota of fermented chicken and seafood products. Furthermore, natural microbiota, such as LAB, have also been used as a natural bio-preservative, an alternative to chemical preservatives. The incorporation of LAB in chicken meat products ensures that the meat products are safe, healthy, and wholesome for consumption without posing any health risks to the consumers. This group of bacteria outgrows the pathogenic organisms owing to their ability to adapt to harsh acidic environments, thereby resulting in the elimination of closely related microbial strains. LAB mainly exert their preservative actions by generating and producing bacteriocins and organic acids. The antimicrobial activity of LAB has been demonstrated in salmon, wherein antimicrobial compounds have been synthesized by LAB, resulting in the modulation of microbiota (Ibrahim et al., 2021). Furthermore, the inhibition of fungal growth and fungicidal activity of LAB against *Aspergillus fumigatus*, *Penicillium expansum*, *Fusarium culmorum*, *Aspergillus niger* has been well investigated in fishery products (Cizeikiene et al., 2013). Therefore, the application of LAB in chicken meat and seafood products allows the elimination of foodborne pathogens and contaminants by producing certain antimicrobial compounds, thus enhancing the shelf-life and storage stability of such products.

Essential oils have been used for the treatment of various diseases and are well-recognized for their potential medicinal value and health benefits since the time memorial. Furthermore, with the increase in the incidence of chronic diseases and metabolic disorders, several investigations have been carried out for the identification of bioactive compounds present in EOs, which are responsible for their biological activity. With progression in time, advances in the extraction techniques of EOs (hydro-distillation, aqueous extraction, solvent extraction, cold and hot pressing, and supercritical fluid extraction process) were investigated for improvement in efficiency and yield. Therefore, the composition and bioavailability of EOs may differ according to the extraction procedure and the method of application in meat products and seafood (Sharma et al., 2019). The bioavailability of EOs depends on many factors including physiological conditions, environmental factors, individual dietary regimens, and others. The majority of the EOs are absorbed from the meat matrix and cross the blood–brain barrier efficiently owing to the hydrophobic nature and smaller size of volatile compounds. Beneficial health effects of EOs and their active compounds are possible through ingestion, inhalation, and absorption through the skin. It has been recognized that EOs containing hydrocarbons (limonene, azulene, cadinene, and myrcene) are associated with antiviral, antibacterial, and hepatoprotective activities, while oxides and esters are related to anti-inflammatory properties (Table 2).

**TABLE 2:** Therapeutic benefits of using essential oils in chicken and seafood.

S. no.	Essential oil	Active component	Properties	Food product	References
1	Chamomile essential oil (*Matricaria recutita*)	Bisabolol, chamazulene	Anti-inflammatory, anti-allergic, decongestant, and antispasmodic	Chicken	Anastasiou et al., 2019
2	Anise essential oil (*Pimpinella anisum*)	Anethole	Antispasmodic, carminative, and diuretic	Chicken filets	Fathi-Achachlouei et al., 2021
3	Nutmeg essential oil (*Myristica Fragrans* Houtt.)	Sabinene, 4-terpineol, myristicin	Anti-microbial, antiparasitic, and analgesic	Ready-to-cook barbecued chicken	Shekarforoush et al., 2014
4	Cedar essential oil (*Juniperus virginiana*)	Limonene	Larvicidal, antifungal, astringent, and decongestant	Chicken breast filet	Kamkar et al., 2021
5	Dill essential oil (*Anethum graveolens L.*)	Carvone	Antispasmodic	Chicken	Vispute et al., 2019
6	Garlic essential oil (*Allium Sativum*)	Diallyl disulfide	Hypoglycemic, antiviral, antifungal, antispasmodic, and antioxidant	Chicken nuggets	Raeisi et al., 2021
7	Clove essential oil (*Syzygium aromaticum* L)	Eugenol	Antiviral, antimicrobial, and carminative	Ready-to-eat chicken sausages	Zubair et al., 2022
8	Cinnamon essential oil (*Cinnamomum zeylanicum*)	Cinnamaldehyde	Antibacterial, antiviral, antifungal, and parasiticide	common carp (*Cyprinus carpio*)	Zhang et al., 2017
9	Sweet orange essential oil (*Citrus sinensis*)	Limonene	Antiseptic, carminative, and tonic	Shrimps	Alparslan et al., 2016
10	Eucalyptus essential oil (*Eucalyptus globulus*)	1,8- cineole	Antimicrobial and antiviral	Fresh chicken meat	Sharafati Chaleshtori et al., 2016
11	Peppermint essential oil (*Menthae piperitae aetheroleum*)	Methanol and menthone	Decongestant, expectorant, antimicrobial, and anesthetic	Fish skin gelatin film	Yanwong and Threepopnatkul, 2015
12	Lavender essential oil (*Lavandula angustifolia*)	Linalool and linalyl acetate	Spasmodic, anti-inflammatory, and antimicrobial	Fish gelatin based composite films	Sun et al., 2021
13	Tea tree essential oil (*Melaleuca alternifolia*)	Terpinene-1-ol-4	Antimicrobial, antiviral, and antispasmodic	Marinated chicken	Wei et al., 2022
14	Lemon essential oil (*Citrus limon*)	Limonene	Digestive tonic carminative, purgative, and metabolism regulator	Rainbow trout meat	Kunova et al., 2021

Phenols and aldehydes present in EOs contribute to antiviral, antipyretic, antimicrobial, vasodilator, and spasmolytic activity. Several EOs, for instance, cinnamon oil, thyme oil, oregano oil, clove oil, and holy basil oil have been used in chicken and seafood products. Polyphenol compounds present in the extract of cinnamon essential oil drastically declined the secretion of TNF-α when given to mice orally for 6 days (Hong et al., 2012), thus indicating the anti-inflammatory property of cinnamon oil. Moreover, cinnamon oil has shown anti-diabetic, anti-cancer, and anti-hypertriglyceridemia potential owing to its biologically active component, cinnamaldehyde. Polyphenols present in EOs are also known to regulate the metabolism of glucose and repair pancreatic β-cells, thus enhancing the islets’ function (Li et al., 2013). Oregano essentials are considered to exert higher antibacterial activity than cinnamon essential oil; however, the major advantage of using the latter in meat and seafood products include the prevention of the development of antimicrobial resistance and their higher efficacy for a longer duration (Almasaudi et al., 2022). The other physical and physiological health benefits of EOs are pain alleviation and memory and mood improvement by rosemary essential oil. Peppermint oil is demonstrated to relive nausea, clear skin conditions, and improve the digestive system, while lavender oil reduces the soreness of muscles and relieves pain. Fast wound healing, mood enhancer, and stress reduction have been documented in one of the earliest known EOs, Frankincense oil (Pirnia et al., 2022).

Apart from antimicrobial activity, the antioxidant activity of chicken meat products was also enhanced, thereby adding to the nutritional value of chicken products (Sharma et al., 2020). The major problem of the chicken meat industry related to oxidative rancidity and subsequent spoilage and deterioration of chicken products has been addressed by the incorporation of blends of EOs (thyme, clove, holy basil, and oregano) in emulsion-based chicken sausage, wherein the antioxidant activity of blend incorporated products was significantly higher (*p* < 0.05) than the control products along with higher total phenolic content (Sharma et al., 2017, 2018). The inclusion of dietary antioxidants reduced the occurrence of certain diseases as these are inversely related to cardiovascular diseases. These mobilize the oxidized low-density lipoproteins in macrophages as of result of which, coronary heart disease incidence is drastically reduced. Furthermore, the antioxidants consumed through meat and meat products also help in declining the pathological changes occurring in the body owing to oxidative stress caused by free radical formation (Wilson et al., 2017). Recent researchers have also demonstrated that the incorporation of EOs in edible packaging film also serves as an antioxidant and antimicrobial preservative for chicken and seafood. In one of the studies, the shelf-life of chicken patties wrapped in an edible film containing a blend of oregano and thyme EOs was enhanced by up to 30 days with no signs of incipient spoilage (Kumar et al., 2018). Though it is argued from time to time that the use of EOs in meat products is an expensive process, and taking into consideration the extraction procedure of essential oil from parts of the plant, the health benefits accrued by the application of EOs in meat products outweigh the disadvantage of cost (Kumar et al., 2017).

## 7 Facts and gaps for the application of LAB and EOs in the meat industry

Essential oils are considered to be comprised of 60–80 components in different proportions, and the components present in higher proportions are responsible for their biological activity and preservative effect on meat products. Studies suggest that EOs containing more aldehydes and phenols exert the most potent effect followed by terpene and ketones containing oils. Therefore, the composition of EOs varies, which mainly contributes to their variation in their mode of action. Furthermore, all the EOs are recognized as hydrophobic in nature, thereby indicating their ability to penetrate cells easily, which, in turn, leads to permeabilization and subsequent death of the cell owing to the depolarization of membranes (Perricone et al., 2015). However, owing to the variety of compounds present in a meat matrix, the major drawback is their interaction with the food matrix. Though the presence of high fat and proteins in meat will interfere with the antibacterial action of EOs in an aqueous phase owing to the protective effect brought by the meat components and their ability to absorb oils, high water and salt concentration in chicken meat products might facilitate the action of EOs. In addition, meat products containing high amount of complex sugar often decreases the efficacy of EOs, which indicates that novel meat products incorporated with EOs should contain a higher amount of simple sugars such as glucose.

The physical state and structure of the food may also affect the efficacy of EOs significantly. For instance, the activity of EOs would always be higher in the product containing more water and liquid as compared to the solid or intact meat piece. This might be due to the diffusion and uniform distribution of EOs in the product. In addition to this, the use of EOs in the packaging films of meat products has also been proven efficacious for their mode of action (Zubair et al., 2021). The major limitation of the application of essential oils in meat products is the perception of a strong aroma, even after cooking, which might decrease their palatability. In this regard, it was proposed to prepare nanoemulsions so that alterations in the organoleptic qualities can be minimized (Xia et al., 2022).

Lactic acid bacteria has played a critical role in the preservation and improvement of the shelf-life of meat and meat products by the production of bacteriocins and organic acids. Several investigations regarding the application of LAB in meat products have been carried out at the lab level; however, reproducing these results at the industrial scale may pose some problems.

## 8 Future perspective

Essential oils contain a variety of compounds that are of biological importance. However, the exact component interacting with meat products still remains uninvestigated in the majority of the products. These studies would further provide insights into the synergistic and antagonistic effect of EOs with particular meat products. Moreover, excessive use of EOs in meat and meat products would not only alter the sensory properties but might cause certain health issues, such as irritation of mucous membranes, respiratory problems, and others. Therefore, the levels and safe doses of essential oils should be optimized and validated using *in vitro* and *in vivo* techniques (Macwan et al., 2016). Finally, more suitable approaches should be devised in order to mask the effect of EOs in meat and seafood products so that the consumer acceptability of these products is not affected. Novel and cost-effective methods and techniques for the targeted delivery or controlled release of EOs should be investigated to protect them from degradation in the gut system and decrease the chance of declined sensory acceptability of the product (Mucha and Witkowska, 2021).

## 9 Conclusion

Chicken meat and seafood are prone to microbial spoilage owing to their neutral pH and higher moisture and fat content. Being highly nutritious, these both require certain preservation methods for their extended shelf-life and maintenance of textural attributes. For long, chemical preservatives have been used; however, these are also associated with toxicological and cancerous problems in consumers; thus natural preservatives, such as LAB and EOs, were explored for their critical role in meat and seafood preservation. Though several investigations and demonstrations have been conducted regarding the application of EOs and LAB in the preservation of chicken meat and seafood products, there are a few limitations that need to be addressed for the utilization of their full potential as a bio-preservative in the area of meat science. Bacteriocins produced by LAB offer major limitations owing to their dependency on the time and temperature of meat products storage, pH of the meat product, and their interaction with the associated microbiota of the food. Another major limitation is the lack of regulations on the use of novel bacteriocins as food additives in meat and meat products.

## Author contributions

HS, HF, and FÖ: conceptualization. HS and HF: methodology, validation, formal analysis, investigation, and writing – original draft preparation. JR: resources. FÖ and JR: data curation, writing – review and editing, and supervision. HS, HF, FÖ, and JR: visualization. All authors have read and agreed to the published version of the manuscript.
